# Investigation of thermal conductivity and rheological properties of nanofluids containing graphene nanoplatelets

**DOI:** 10.1186/1556-276X-9-15

**Published:** 2014-01-13

**Authors:** Mohammad Mehrali, Emad Sadeghinezhad, Sara Tahan Latibari, Salim Newaz Kazi, Mehdi Mehrali, Mohd Nashrul Bin Mohd Zubir, Hendrik Simon Cornelis Metselaar

**Affiliations:** 1 Advanced Material Research Center, Department of Mechanical Engineering, University of Malaya, Kuala Lumpur 50603, Malaysia; 2 Department of Mechanical Engineering, University of Malaya, Kuala Lumpur 50603, Malaysia

**Keywords:** Graphene nanoplatelets, Nanofluids, Thermal conductivity, Rheology, Stability

## Abstract

In the present study, stable homogeneous graphene nanoplatelet (GNP) nanofluids were prepared without any surfactant by high-power ultrasonic (probe) dispersion of GNPs in distilled water. The concentrations of nanofluids were maintained at 0.025, 0.05, 0.075, and 0.1 wt.% for three different specific surface areas of 300, 500, and 750 m^2^/g. Transmission electron microscopy image shows that the suspensions are homogeneous and most of the materials have been well dispersed. The stability of nanofluid was investigated using a UV-visible spectrophotometer in a time span of 600 h, and zeta potential after dispersion had been investigated to elucidate its role on dispersion characteristics. The rheological properties of GNP nanofluids approach Newtonian and non-Newtonian behaviors where viscosity decreases linearly with the rise of temperature. The thermal conductivity results show that the dispersed nanoparticles can always enhance the thermal conductivity of the base fluid, and the highest enhancement was obtained to be 27.64% in the concentration of 0.1 wt.% of GNPs with a specific surface area of 750 m^2^/g. Electrical conductivity of the GNP nanofluids shows a significant enhancement by dispersion of GNPs in distilled water. This novel type of nanofluids shows outstanding potential for replacements as advanced heat transfer fluids in medium temperature applications including solar collectors and heat exchanger systems.

## Background

Processes of energy transport have been integrated in a wide range of areas, such as in industry, oil and gas, and electricity. In the past decades, ethylene glycol, water, and oil were used as conventional fluids in heat exchanger systems. However, improvement of these conventional heat transfer fluids, particularly thermal conductivity, has become more and more critical to the performance of energy systems [1]. Choi and Eastman [2] have introduced the term nanofluids referring to fluids containing dispersed nanosized particles having large thermal conductivity enhancement. In spite of the attention received by this field, uncertainties concerning the fundamental effects of nanoparticles on thermophysical properties of solvent media remain [3]. Thermal conductivity is the property that has catalyzed the attention of the nanofluid research community [4]. A lot of research has been devoted to improve the thermal properties of these fluids by adding a small quantity of a highly thermal conductive solid at concentrations ranging from 0.001 to 50 wt.% of the various nanomaterials including oxide [5], nitride [6], metal [7], diamond [8], carbon nanotube [9], carbon fiber [10], carbon black, graphene oxide [11], graphene [12], graphite flake [13], and hybrid [14] with different shapes (particle, disk, tube, sheet, fiber, etc.) [4,15,16]. Nanofluids have many applications in the industries since materials of nanometer size have unique chemical and physical properties and the thermal conductivity of nanofluids with smaller size of nanoparticles is larger than the those of bigger sizes at specific concentrations [17].

Recently, a significant number of studies have been conducted on the use of carbon-based nanostructures like carbon nanotubes [18], single-wall carbon nanotubes [19], multiwall carbon nanotubes [20], graphite [21], graphene oxide [22], and graphene [23] to prepare nanofluids. Recent studies reveal that graphene has a very high thermal conductivity, so it is obvious that graphene nanofluid would show a higher thermal conductivity enhancement compared to other nanoparticles. Graphene, a single-atom-thick sheet of hexagonally arrayed sp^2^-bonded carbon atoms, has attracted much attention since its discovery by Novoselov et al. [24]. Graphene nanoplatelets are two-dimensional (2D) with an average thickness of 5 to 10 nm and a specific surface area of 50 to 750 m^2^/g; they can be produced at different sizes, from 1 to 50 μm. These interesting nanoparticles, including short stacks of platelet-shaped graphene sheets, are identical to those found in the walls of carbon nanotubes but in planar form [25]. Graphene nanoplatelets (GNPs) have drawn a lot of interest due to their excellent electrical conductivity and high mechanical properties; the in-plane thermal conductivity of GNPs is reported to be as high as 3,000 to 5,000 W/m∙K [26]. Further, as this is a 2D material, the heat transfer properties are expected to be much different from the zero-dimensional nanoparticles and one-dimensional carbon nanotubes. Moreover, since GNP itself is an excellent thermal conductor, graphene-based nanofluids are normally expected to display a significant thermal conductivity enhancement [27]. Graphene nanoplatelets are also offered in granular form which could be dispersed in water, organic solvents, and polymers with the right choice of dispersion aids, equipment, and techniques.

In this paper, an attempt is made to prepare aqueous suspensions of stable homogeneous GNP nanofluids by high-power ultrasonication. It is difficult to disperse GNPs for high mass percentages without using any dispersion aids; in this work, stable homogeneous GNP nanofluids were prepared for the first time without the use of any surfactant, and their thermophysical properties were measured at temperatures below 100°C. Stabilization mechanisms of dispersions are analyzed by UV-visible (vis) spectrophotometry and zeta potential measurements to quantitatively characterize the colloidal stability of the GNP dispersions. It is expected that the final results can provide a guideline for selecting ideal dispersants. The present report contains results on thermal conductivity, viscosity, and stability of three different specific surface areas (300, 500, and 750 m^2^/g) at different concentrations (by weight percentage) of the mixture of GNPs and distilled water as base fluid. Results have been discussed to identify the mechanisms responsible for the observed thermal conductivity and viscosity enhancement in GNPs prepared at different concentrations (0.025, 0.05, 0.075, and 0.1 wt.%) of the mixture of GNPs and distilled water. The feasibility of the GNP nanofluids for use as innovative heat transfer fluids in medium temperature heat transfer systems has been demonstrated.

## Methods

### Materials

GNPs have special properties dependent on the number of layers, such as saturable absorption, linear monochromatic optical contrasts, and electric field-assisted bandgaps, which are not found in previously produced materials. These materials (Grade C, XG Sciences, Inc., Lansing, MI, USA) were used for the preparation of nanofluids. Each grade contains particles with a similar average thickness and specific surface area. Grade C particles have an average thickness of a few nanometers and a particle diameter of less than 2 μm. The average specific surface areas are 300, 500, and 750 m^2^/g, and all specifications are shown in Table 1.

**Table 1:** Nanoparticle specification

**Property**	**Specification**
Particle	GNPs
Color	Black granules/powder
Carbon content	>99.5
Bulk density	0.2 to 0.4 g/cm^3^
Relative gravity	2.0 to 2.25 g/cm^3^
Specific surface area	300, 500, and 750 m^2^/g
Particle diameter	2 μm
Peak in UV–vis spectrophotometer	265 to 270 nm
Thickness	2 nm
Thermal conductivity	
Parallel to surface	3,000 W/m∙K
Perpendicular to surface	6 W/m∙K
Electrical conductivity	
Parallel to surface	10^7^ S/m
Perpendicular to surface	10^2^ S/m

### Nanofluid preparation

**Figure 1:**
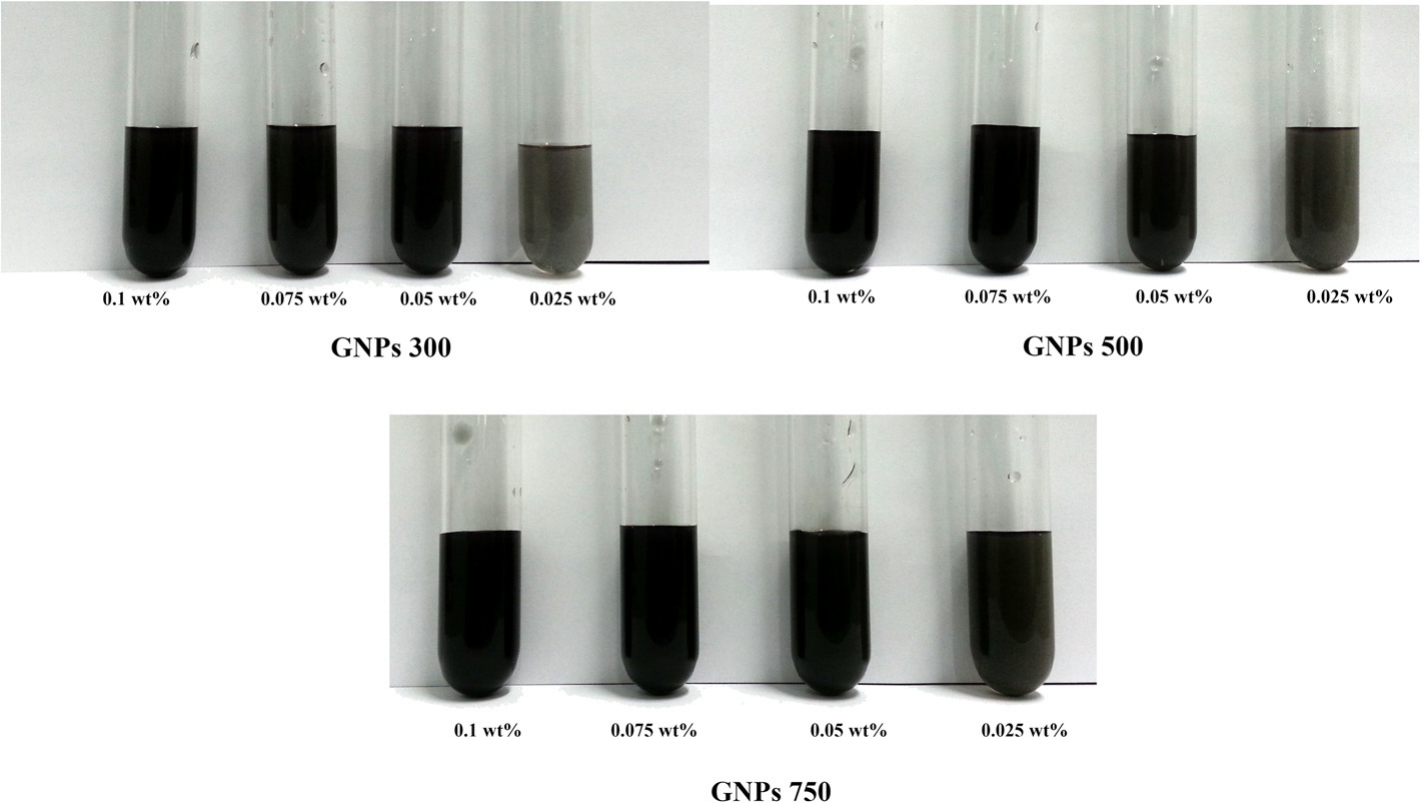
Photo of GNP nanofluids after 600 h of storage time.

Dispersion of nanoparticles into the base fluid is an important process requiring special attention. The prepared nanofluid should be an agglomerate-free stable suspension without sedimentation for long durations. Graphene nanoplatelets are offered in granular form that is soluble in water with the right choice of dispersion aids, equipment, and techniques. The graphene nanoplatelets were dispersed in distilled water using a high-power ultrasonication probe (Sonics Vibra Cell, Ningbo Kesheng Ultrasonic Equipment Co., Ltd., Ningbo, China) having a 1,200-W output power and a 20-kHz frequency power supply. The concentrations of nanofluids were maintained at 0.025, 0.05, 0.075, and 0.1 wt.% for specimens of three average specific surface areas of 300, 500, and 750 m^2^/g. The stable homogeneous GNP nanofluids were prepared for the first time without using any surfactant. Photos of three typical samples of GNP nanofluids at a concentration after 600 h are shown in Figure 1.

### Analysis methods

Detailed microstructures were further examined under a transmission electron microscope (TEM; TEM-LIBRA 120, Carl Zeiss, Oberkochen, Germany). The rheological behavior of the nanofluids of different weight percentage of graphene nanoplatelets was measured using an Anton Paar rheometer (Physica MCR 301, Anton Paar GmbH, Graz, Austria), which had recorded the viscosity and shear rate for different temperatures. Electrical conductivity and zeta potential of the nanofluids were measured using Zetasizer Nano (Malvern Instruments Ltd., Malvern, UK). A transient heated needle (KD2 Pro, Decagon Devices, Inc., Pullman, WA, USA) was used to measure the thermal conductivity with 5% accuracy at constant temperature. The thermal conductivity measurements were repeated ten times, and the average values were reported. Light transmission of all samples was measured with a Shimadzu UV spectrometer (UV-1800, Shimadzu Corporation, Kyoto, Japan) operating between 190 and 1,100 nm. The nanofluid solution was diluted with distilled water to allow sufficient transmission while each measurement was repeated three times to achieve a better accuracy.

## Results and discussion

### Morphology of GNP dispersions

**Figure 2:**
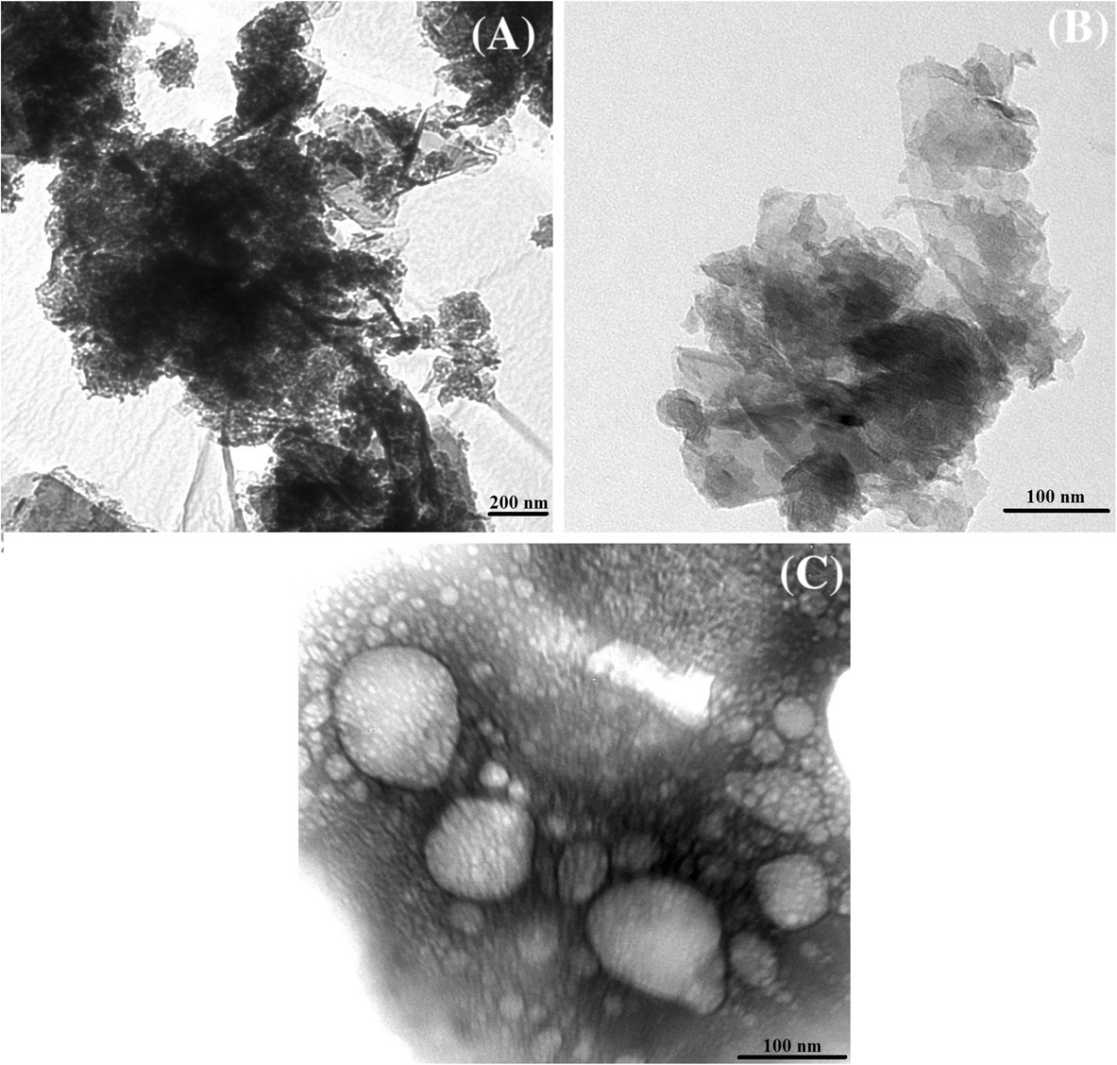
**TEM images of GNP nanoparticles. (A)** GNP 300, **(B)** GNP 500, and **(C)** GNP 750.

A drop of diluted solution was placed onto a carbon-coated copper grid, air-dried, and observed under TEM. Figure 2 shows the image of dried GNP suspensions with different specific surface areas. For the GNPs, the sheet-like structure with a lateral size at the micrometer length scale has been well captured as shown in Figure 2. Notably, the GNPs show good flexibility as proven by the folded and/or rolled parts. This indicates that each of the GNP sheets only contains a very limited number of graphene layers, which is consistent with the parameter provided by the manufacturer. When GNPs were dispersed by ultrasonic treatment, the lateral size of GNPs was decreased. The edges of GNP layers are clearly seen as straight lines. At higher specific surface area, the GNP size becomes smaller. The sonication process tends to break the flake: longer sonication time improves the exfoliation degree; further sonication is advantageous from the aspect of dispersion and colloidal stability.

### Stability

#### Stability investigation with UV–vis spectroscopy

UV–vis spectrophotometer analysis is a convenient approach to characterize the stability of colloids quantitatively. Light absorbency ratio index can be calculated using the Beer Lambert law as shown in Equation 1:

(1) $$A=-\log\left(\frac{I}{I_{i}}\right)=\mathrm{\epsilon bc}$$

**Figure 3:**
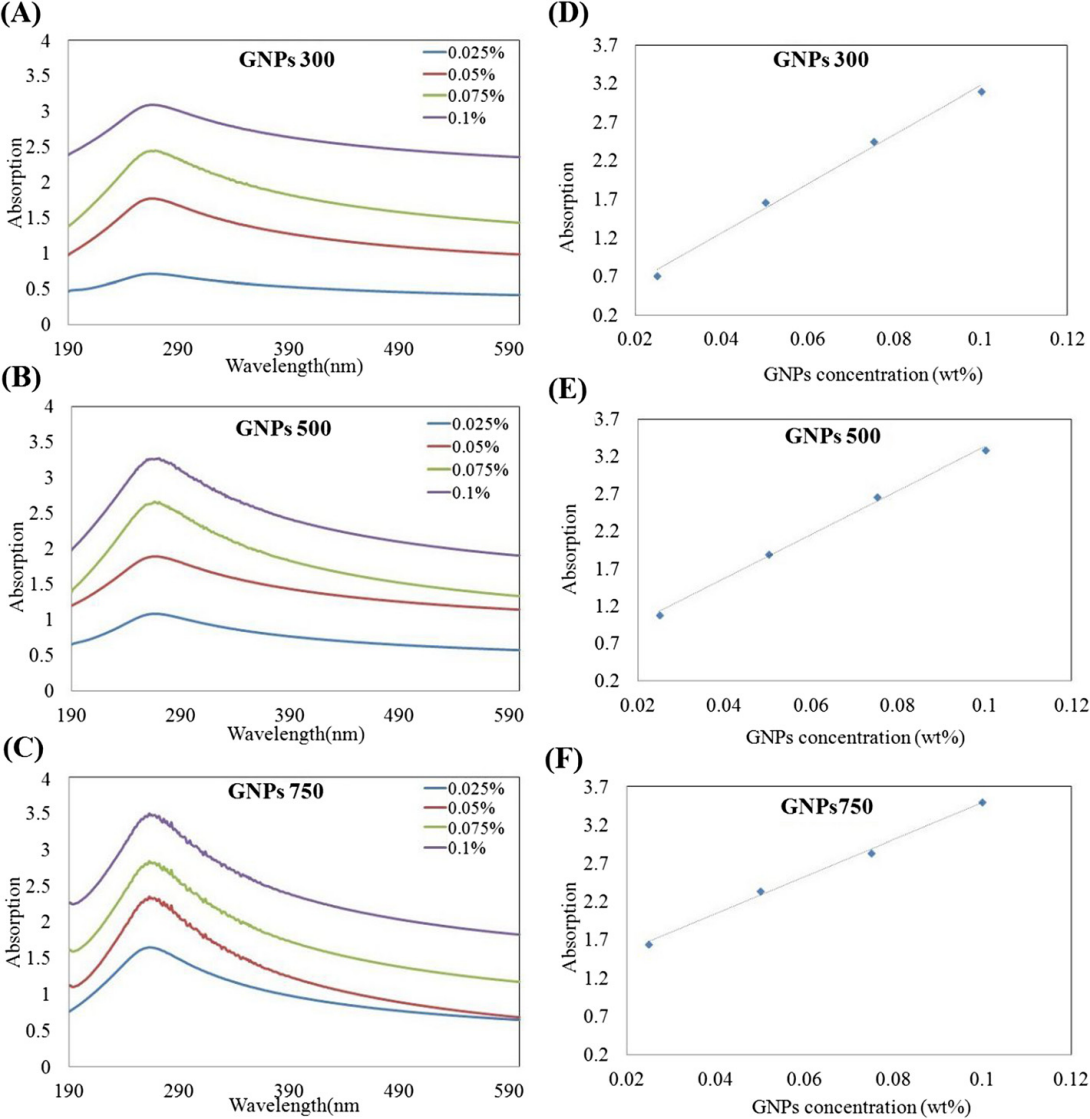
**UV–vis spectrophotometers of GNPs nanofluids. (A, B, C)** UV–vis spectrophotometer of GNPs nanofluids at different concentrations and wavelength and **(D, E, F)** absorption values of GNPs dispersed in distilled water at different concentrations.

Equation 1 shows that at fixed molar optical path and absorptivity, the absorbency is relative to the weight percentage of the particles inside the suspension. Figure 3A,B,C shows the UV–vis spectrophotometry images of the GNP dispersions at different concentrations with different specific surface areas. The UV–vis spectrum of GNP dispersion in distilled water is featureless with a monotonic decrease in absorbance with increasing wavelength, except below 320 nm where a strong absorption band is observed, which scales with GNP concentration but is less independent of GNP specific surface area. Moreover, the absorbance of GNPs decreases from 0.1 to 0.025 wt.%; it should be known that the increasing amount of dispersed GNPs will increase the absorbance that refers to the better nanofluid dispersion. From the results, it can be seen that by increasing the specific surface area of GNPs, the absorption value of *λ*_max_ increased for the same concentration, which means that a higher specific area gives a better GNP dispersion. As can be seen in Figure 3, the absorption value of *λ*_max_ at 280 nm shows no visible changes; the GNP nanofluids are considered to be stable. The suddenly decreased absorption value indicates that the GNP nanoparticles in the nanofluids start to aggregate and deposit. As shown in Figure 3D,E,F, there is a good linear relationship between the absorbance and the concentration of GNPs, which satisfies Beer's law and indicates that GNP sheets were dispersed well in the base fluid.

**Figure 4:**
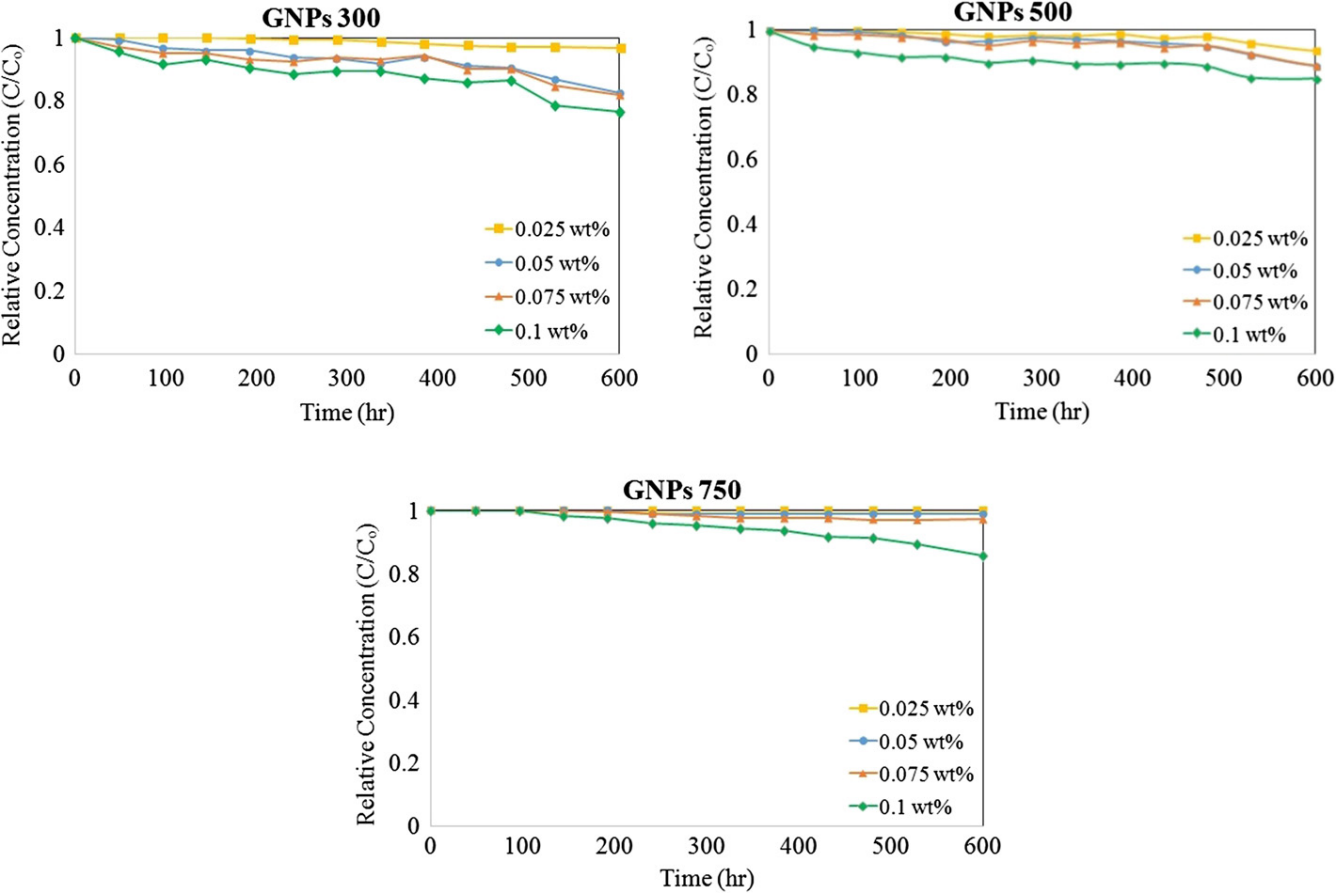
Relative particle concentration of nanofluids with sediment time.

Figure 4 shows colloidal stability of aqueous GNPs of nanofluids as a function of sedimentation time. From the results, it can be seen that the relative concentration for the same specific surface area and different concentrations was decreased due to slight agglomeration and precipitation by the increasing concentration. The best relative concentration of nanofluid compared with the fresh one is for GNP 750, which has a concentration of 0.025 wt.%, because of the higher specific surface area and lower concentration of GNPs. As a result, specific surface area of GNPs shows a very effective influence on the stability of the nanofluid.

The rate of sedimentation after 600 h is different among these 12 samples as different concentrations and specific surface areas are imposed. This rate is changing as the lowest precipitation rate appears from 1% by GNP 750 (0.025 wt.%) to the highest of 24% by GNP 300 (0.1 wt.%). These results show that different concentrations and specific surface areas affect the rate of sedimentation as well as properties, which agree well with the results of previous studies [28].

#### Stability investigation with zeta potential

**Figure 5:**
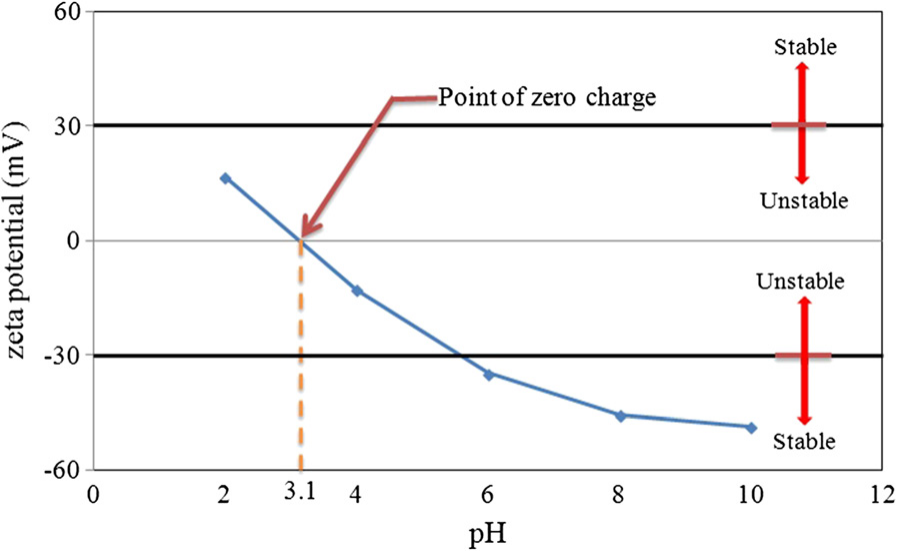
**Zeta potential values of GNP (750 m**^**2**^**/g) nanofluids as a function of pH value.**

The measurement of the zeta potential has carried out the electrophoretic behavior and additional details to comprehend the dispersion behavior of GNPs in water. Zeta potential values of GNP nanofluid were obtained for various specific areas. Figure 5 demonstrates the changes of zeta potential for GNP 750 suspensions as a function of pH values. In the GNP suspension, while using water as a base fluid, the GNPs tend to be positively charged before pH 3 and negatively charged within the entire pH ranges after pH 3. At approximately pH 10, the absolute value of zeta potential will be at maximum, while the maximum excess is 50 mV. The nanofluids which have a measured zeta potential above +30 mV or below −30 mV are having good stability [29]. It implies that the force of electrostatic repulsion between GNPs is sufficient to get over the attraction force between particles. Higher electrostatic force may also cause to form much more free particles by improving particle-particle distance, in order that the distance exceeds the hydrogen bonding range between particles and further decreases the chance of particle coagulation and settling. The pH value of prepared nanofluids was measured at about pH 8 while zeta potential value appears to be 31.8, 40.9, and 45.7 mV for GNPs at 300, 500, and 750 m^2^/g, respectively. The inclination is that the zeta potential values demonstrate an enhancement for higher specific surface areas of GNPs. This phenomenon suggests that the GNPs nanofluid with higher specific surface areas might have better stability.

### Rheological behavior of GNPs

**Figure 6:**
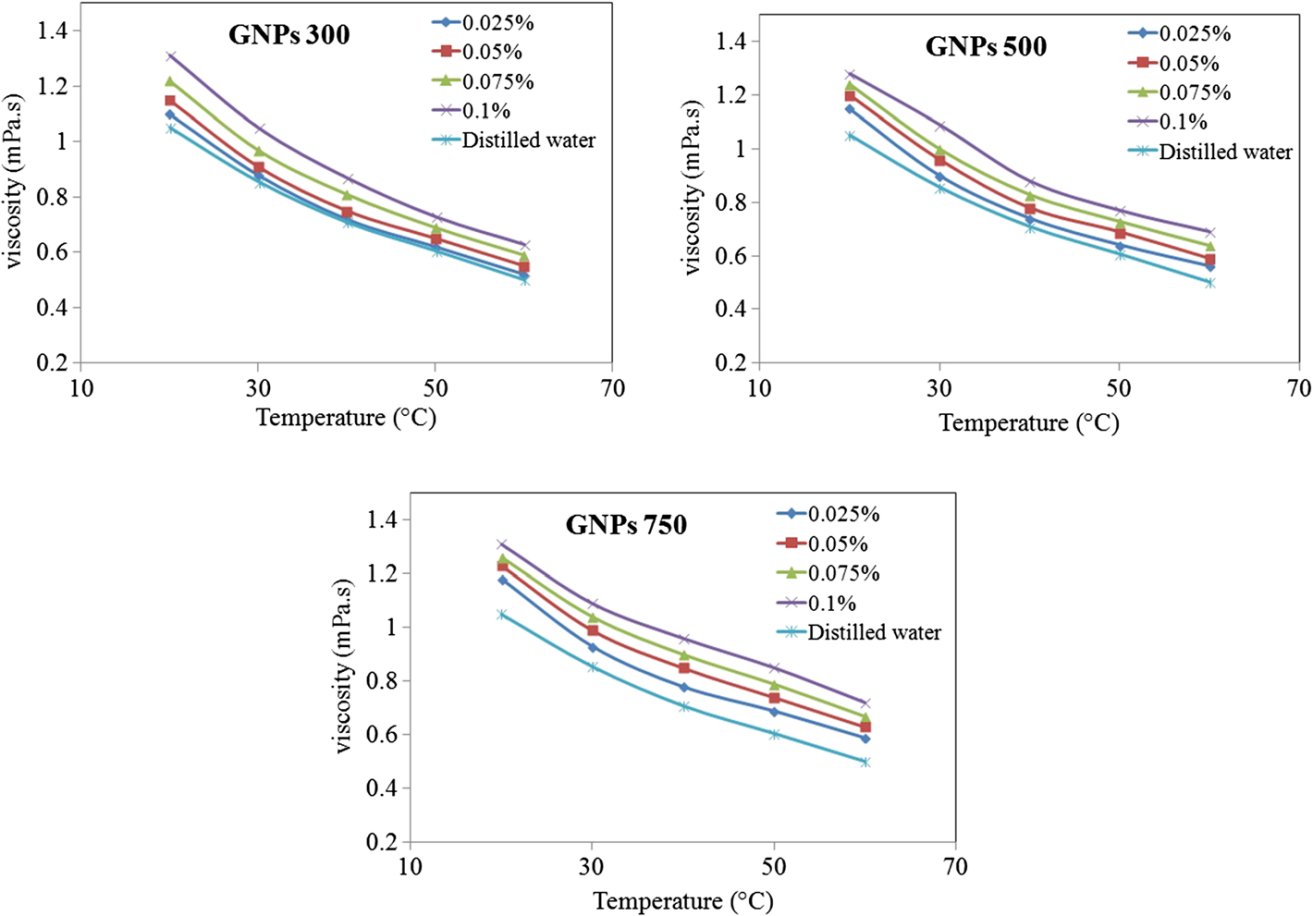
Viscosity versus concentration at various temperatures and constant shear rates.

Viscosity of nanofluids is one of the most critical parameters, which determines the quality of heat transfer fluid. Similar to simple fluids, temperature is the main effective parameter on viscosity of nanofluids. As expected, distilled water exhibits a Newtonian behavior within the shear rate range investigated. The viscosity value of distilled water was 1.034, which closely matches with its theoretical values at 20°C. The relative deviation is less than 2.5%. This is of the same order of magnitude as the experimental uncertainty. Figure 6 reports the viscosity at a high shear rate of 500/s for different concentrations and specific surface areas as a function of all tested temperatures. While nanofluids and base fluids are strongly dependent on temperature, it is also observed in Figure 6 that the viscosity was decreased for higher temperatures. This is expected due to the weakening of the interparticle and intermolecular adhesion forces, and similar trends have also been observed in almost all other varieties of nanofluids. It can be clearly seen that viscosity increased for higher concentrations of GNPs and that the viscosity of nanofluid improved by 44% compare to the viscosity of the base fluid for 0.1 wt.% of GNPs. This can be realized in such a way that once the concentration increases, the nanoparticles make an agglomeration within the suspension. This consequently results in the increase of internal shear stress in nanofluid because of the greater force needed for dissipating the solid element of the dispersion and hence an increase in viscosity.

**Figure 7:**
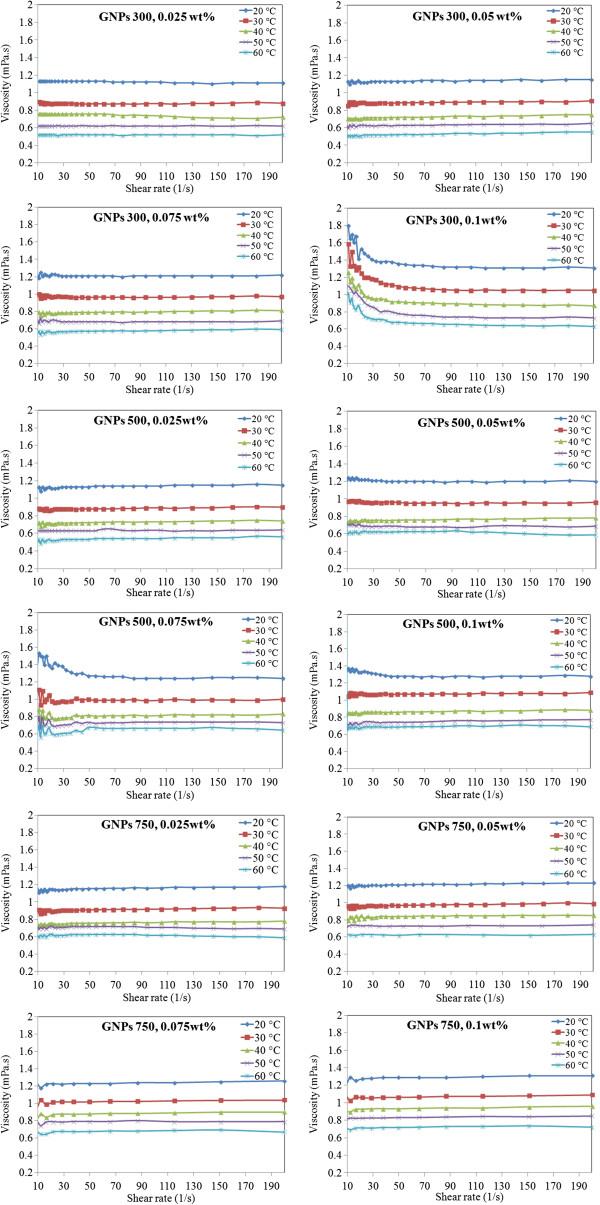
Plots of viscosity versus shear rate at various concentrations and temperatures.

In order to determine the rheological behaviors of GNP nanofluids, the viscosity of aqueous GNPs versus shear rate was measured at the temperature range of 20°C to 60°C, and the results are shown in Figure 7. The viscosity of distilled water decreases exponentially as a function of shear rate which indicates its shear thinning (pseudoplastic) behavior. Following the trend of water, the samples of GNP nanofluid also exhibit the shear thinning property. The cause of this non-Newtonian shear thinning can be explained generally as follows. At low shear rates, as the spindle rotates in the fluid, the structure of the fluid molecules changes temporarily and gradually aligns themselves in the direction of increasing shear; it produces less resistance and hence a reduction in viscosity. When the shear rate is high enough, the maximum amount of possible shear ordering is attained, and the aggregates are broken down to smaller sizes, decreasing the friction and hence the viscosity [30]. If we increase the shear rate further, it will not make any alteration on the viscosity. Due to small size and large surface area of the nanoparticle, there is a possibility for structuring at low shear rates and a deformation and restructuring at high shear rates. Hence, nanofluid also follows the same trend. It is observed at all temperatures that the shear thinning property is more pronounced at higher concentrations. This points out that at low concentrations, the nature of base fluid plays a major role in shear thinning, but at higher concentrations, there is a significant contribution from the interaction between the nanoparticle and fluid.

The results indicate that prepared nanofluids are suitable to use at elevated temperatures. By increasing the temperature, thermal movement of molecules and Brownian motion intensify and intramolecular interactions become weakened. In addition, rheological test on nanofluids revealed that higher concentration increases the viscosity; however, other investigated parameters such as temperature and specific surface areas have an important influence on the viscosity behavior of nanofluids.

### Thermal conductivity

**Figure 8:**
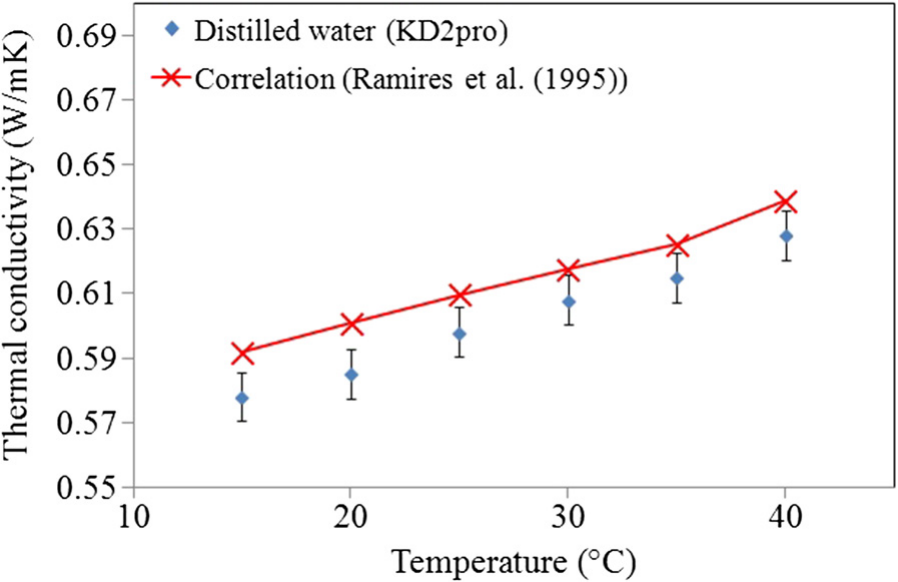
Comparison between distilled water data from KD2pro and previous data.

**Figure 9:**
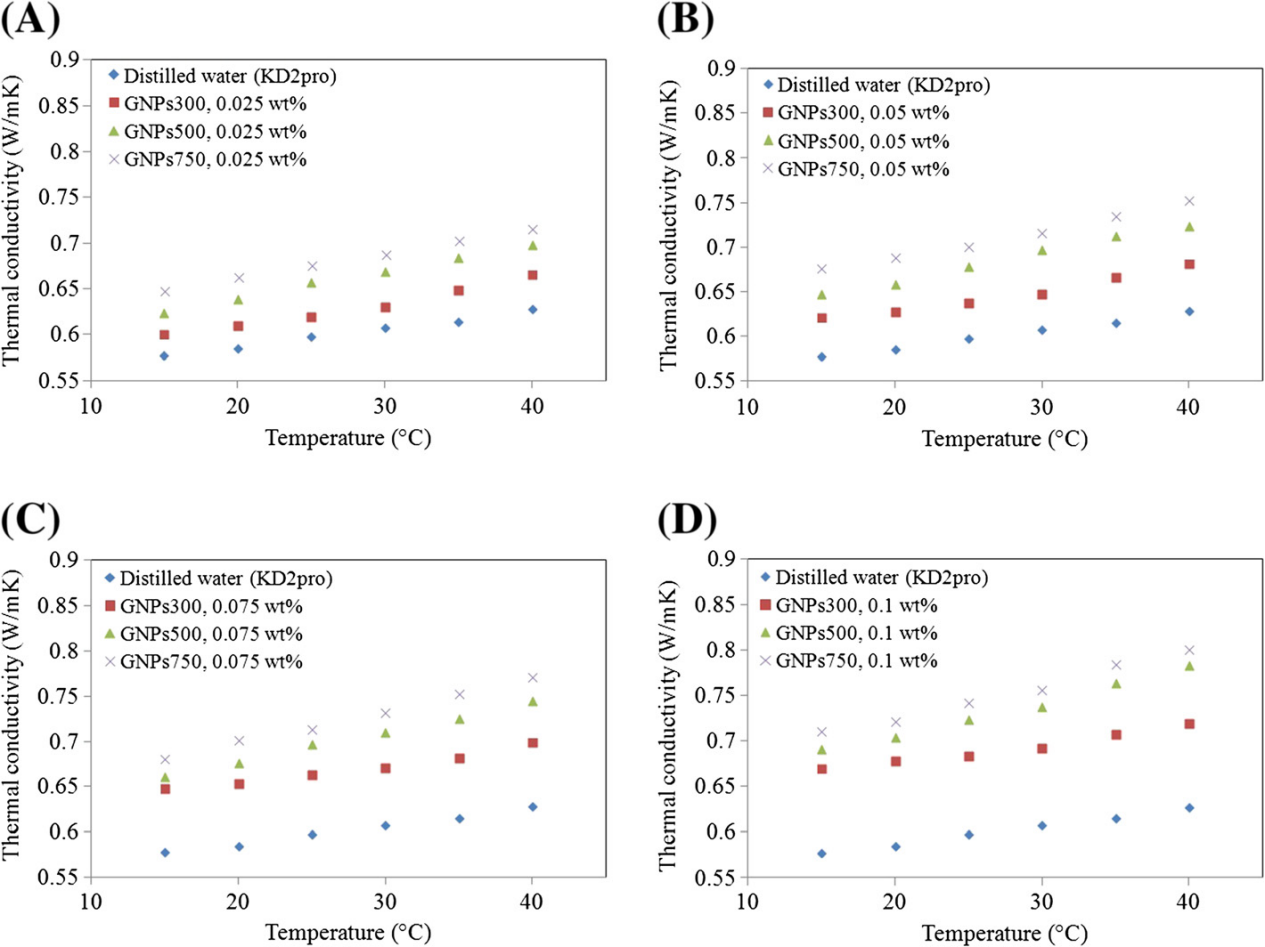
**Thermal conductivity of GNP nanofluids by changing of temperature with different GNP concentrations. (A)** 0.025 wt.%, **(B)** 0.05 wt.%, **(C)** 0.075 wt.%, and **(D)** 0.1 wt.%.

The development of high-performance thermal systems has increased the interest on heat transfer enhancement techniques where heat transfer fluids play an important role in developing efficient heat transfer equipment. Thermal conductivity measurements in this work were done based on the THW method, and the analyzer device has a 5% accuracy over 5°C to 40°C temperature range. In the present study, the calibration tests for distilled water was verified by previous data [5,17,31], and the results are obtained within 2% to 4% accuracy as demonstrated in Figure 8. The thermal conductivity of the nanofluids with various concentrations of GNPs was measured, and results are shown in Figure 9 for the temperature ranging 15°C to 40°C.

**Figure 10:**
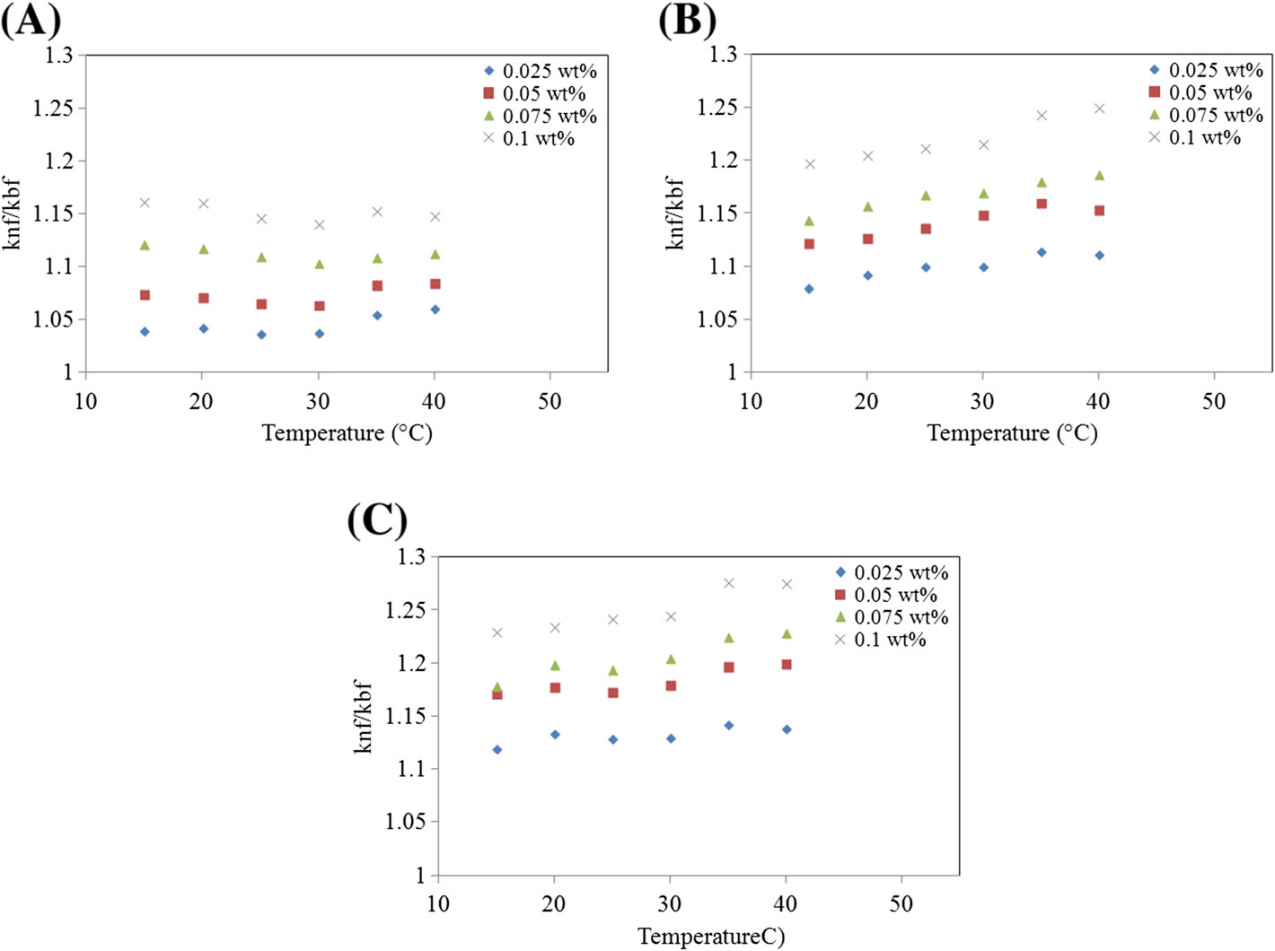
**Thermal conductivity ratios of GNPs with different concentrations and specific surface areas. (A)** GNP 300, **(B)** GNP 500, and **(C)** GNP 750.

From the results, it can be seen that the higher thermal conductivity belongs to the GNPs with higher specific surface area as well as for higher particle concentrations. The standard thermal conductivity models for composites, such as the Maxwell model and the Hamilton-Crosser model, and the weakness of these models in predicting the thermal conductivities of nanofluids led to the proposition of various new mechanisms. The Brownian motion of nanoparticles was indicated by several authors [32,33] as a prime factor for the observed enhancement. However, it is now widely accepted that the existence of a nanolayer at the solid–liquid interface and nanoparticle aggregation may constitute major contributing mechanisms for thermal conductivity enhancement in nanofluids. The liquid molecules close to particle surfaces are known to form layered structures and behave much like a solid. Figure 10 shows the thermal conductivity ratio for different GNPs at different specific surface areas for temperatures between 15°C and 40°C. The linear dependence of thermal conductivity enhancement on temperature was obtained. From Figure 10, a similar trend of thermal conductivity enhancement is observed when concentration and temperature are increased. The enhancement in thermal conductivity for GNP 300 was between 3.98% and 14.81%; for GNP 500, it was between 7.96% and 25%; and for GNP 750, it was between 11.94% and 27.67%. It was also observed that for the same weight percentage and temperature, GNP 750-based nanofluid presents higher thermal conductivity values than those of the other base fluids with GNPs that had lower specific surface area.

**Figure 11:**
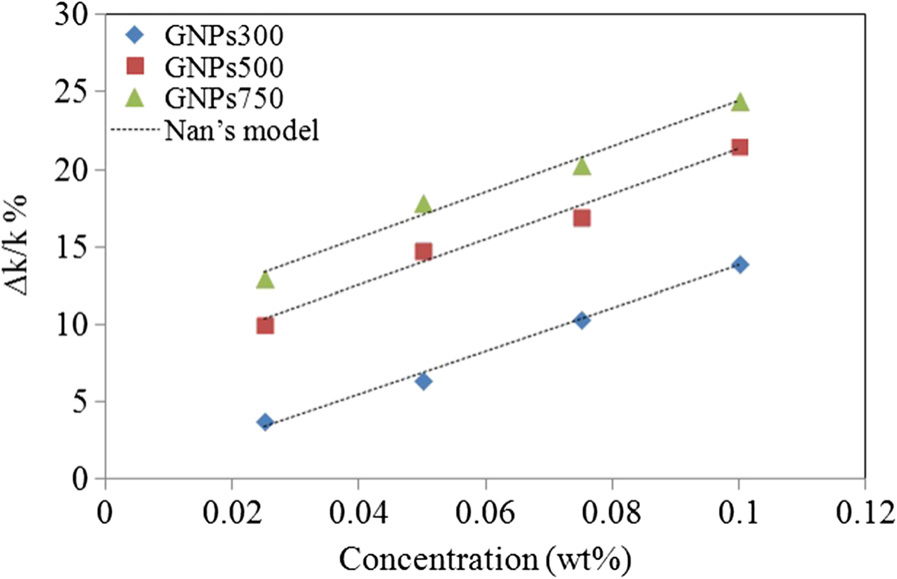
Thermal conductivity enhancement based on Nan's model and experimental results at 30°C.

It is clear that after the nanoparticle materials as well as the base fluid are assigned, the effective thermal conductivity of the nanofluid relied on concentration (*φ*) and temperature. Consequently, it is apparent that the thermal conductivity and dimension (thickness) of the interfacial layer have important effects on the enhanced thermal conductivity of nanofluids. The typical theoretical models that have been developed for thermal conductivity of nanoparticle-suspended fluids considered only thermal conductivities of the base fluid and particles and volume fraction of particles, while particle size, shape, and the distribution and motion of dispersed particles are having significant impacts on thermal conductivity enhancement. Therefore, the experimental results could not be compared with the correlated values of theoretical models. Scientists from different organizations throughout the world accomplished a benchmark research on the thermal conductivity of nanofluids, and the results indicated that the experimental data were in good agreement when Nan's model is used. According to Nan's model, the thermal conductivity of the nanofluid can be calculated as follows:

(2) $$k_{\mathrm{nf}}=k_{\mathrm{bf}}\frac{3+\phi \left[2\beta_{11}\left(1-L_{11}\right)+\beta_{33}\left(1-L_{33}\right)\right]}{3-\phi \left(2\beta_{11}L_{11}+\beta_{33}L_{33}\right)}$$

where *L*_*ii*_ and *ϕ* are the geometrical factor and the volume fraction of particles, respectively. *β*_*ii*_ is defined as

(3) $$\beta_{\mathrm{ii}}=\frac{k_{p}-k_{\mathrm{bf}}}{k_{\mathrm{bf}}+L_{\mathrm{ii}}\left(k_{p}-k_{\mathrm{bf}}\right)}$$

where *k*_p_ is the thermal conductivity of the particles. For GNPs, the aspect ratio is very high, so *L*_11_ = 0 and *L*_33_ = 1. It should be mentioned that the thermal conductivity determined here by Nan's model has taken the matrix additive interface contact resistance into consideration. In Equation 2, the predicted thermal conductivity of composite is sensitive to the small change of the nanoparticles' thermal conductivity. Additionally, the theoretical calculation established that the thermal conductivity of graphene can be influenced by the dimensions, edge roughness, and defect density. Figure 11 shows the thermal conductivity enhancement of GNP nanofluids as a function of loading at a constant temperature of 30°C. From the results, it can be clearly seen that experimental results can be validate using Nan's model. Furthermore, the comparison between carbon-based nanofluids in most recent works is shown in Table 2.

**Table 2:** Thermal conductivity enhancement of recent nanofluids in literature

**Base fluid**	**Concentration (wt.%)**	**Dispersant + base fluid**	**Maximum enhancement (%)**	**Reference**
MWNTs	0.60	DW	34	[34]
Graphite	0.5	DW + PVP	23	[35]
GO	12	EG	61	[11]
GNP 300	0.1	DW	14.8	Present study
GNP 500	0.1	DW	25	Present study
GNP 750	0.1	DW	27.6	Present study

MWNTs, multiwall carbon nanotubes; GO, graphene oxide; DW, distilled water; EG, ethylene glycol; PVP, polyvinylpyrrolidone.

Based on the results in Table 2, it is outstandingly evident that GNP nanofluids provide a significant thermal conductivity enhancement compared to those of other works when they have higher concentrations of nanoparticles. From these results, it can be seen that the use of low concentration of GNPs can achieve acceptable thermal conductivity enhancement for medium-temperature applications including solar collectors and heat exchanger systems.

### Electrical conductivity analysis

**Figure 12:**
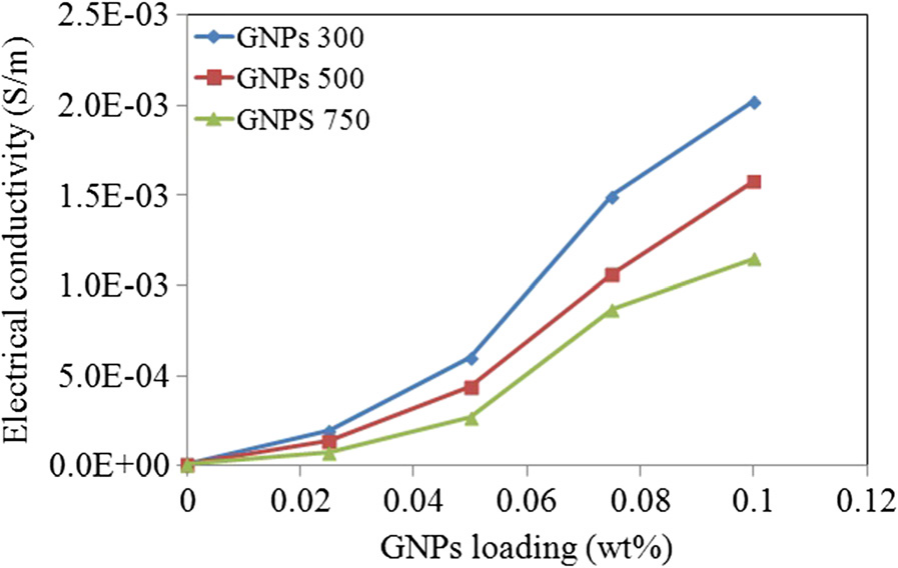
**Electrical conductivity (*σ*) of GNPs.**

Though important, the electrical conductivity of nanofluids has not yet been widely studied as compared to thermal conductivity. The electrical conductivity of a suspension can either increase or decrease depending on the background electrolyte, particle size, particle loading, and charge of the particle. The electrical conductivity (*σ*) of water is related to the temperature and increases by 2% to 3% for each 1°C increase (typical electrical conductivity of distilled water at 25°C is about 5.5 × 10^−6^ S/m). The electrical conductivity of the nanofluids was obtained at 25°C, and the results are presented in Figure 12. For nanofluids with GNP 300, electrical conductivity increases to about 21 μS/cm for a mass percentage of 0.1%, while electrical conductivity of water is about 2 μS/cm. The enhancement in electrical conductivity was determined by the formula [((*σ* − *σ*_0_) × 100)/*σ*_0_] where ‘*σ*_0_’ refers to the electrical conductivity of base fluid and ‘*σ*’ that of nanofluid. The maximum enhancement of around 950% was observed at 25°C which was for GNP 300. Through the results, it could be seen that electrical conductivity was enhanced by increasing mass percentage along with decreasing specific surface area.

## Conclusions

Stability and thermophysical properties of GNP nanofluids have been studied systematically, and the following conclusions could be drawn from the results. The nanofluids of GNPs prepared by ultrasonication were stable for a long period of time. Detailed measurements were carried out to determine the effect of particle mass concentration, specific surface area, and temperature on the thermophysical properties of GNP nanofluid. The rate of increase in thermal conductivity of nanofluids is found to be very significant at higher specific surface area of GNPs due to factors like stability, homogeneity, and rate of agglomeration. The maximum percentage of enhancement in thermal conductivity was obtained at 27.64% for the loading of 0.1 wt.% of GNP 750 at 35°C. The shear rate of nanofluids increased when higher specific surface areas and concentration of GNPs were used. It can be inferred that GNP nanofluids could be a useful and cost-effective material for heat transfer applications along with the development of a facile approach to a large-scale production of aqueous GNP dispersions without any surfactant stabilizers.

## Nomenclature

*A* absorbency

*b* optical path (cm)

*c* molar concentration (mol/dm^3^)

GNPs graphene nanoplatelets

*I* transmitted light intensity

*I*_*i*_ incident light intensity

*k*_*bf*_ thermal conductivity of base fluid

*k*_*nf*_ thermal conductivity of nanofluids

*k*_*p*_ thermal conductivity of the particle

TEM transmission electron microscopy; wt.% weight percentage

2D two-dimensional

*ϕ* particle volumetric fraction

ϵ molar absorptivity, L (mol^−1^ cm^−1^) 

## Competing interests

The authors declare that they have no competing interests.

## Authors' contributions

The manuscript was written through the contributions of all authors MM, ES, STL, SNK, MM, MNMZ, and HSCM. All authors read and approved the final manuscript.
